# Hosting and Human Rights: The Summer Olympics in the Twenty-First Century

**DOI:** 10.3389/fspor.2022.779522

**Published:** 2022-04-25

**Authors:** MacIntosh Ross, Michael McDougall

**Affiliations:** ^1^School of Kinesiology, Western University, London, ON, Canada; ^2^Department of Psychology, Keystone College, La Plume, PA, United States

**Keywords:** Olympics, world order, hegemony, International Relations, human rights, neoliberalism, celebration capitalism

## Abstract

During the twenty-first century, Summer Olympic Games have been used to distract from, justify and push through acts of increased securitization, surveillance, and displacement of the host city populace. Situating sport within the field of International Relations, we outline these civil and human rights intrusions across successive Games. From Sydney 2000 to Rio de Janeiro 2016, we explicate the consequences, contestedness, and evolution of repressive techniques applied at each Games using theories of hegemony espoused by Antonio Gramsci, Robert W. Cox, and Raymond Williams, among others. In doing so, we demonstrate how the International Olympic Committee (IOC), their partners and host cities are wedded in a symbolic and symbiotic courtship that manufactures local consent for and normalizes human right infringements; simultaneously providing the architecture for the spread and imposition of neoliberal order on the citizenry, while masking the damage done by and through the Olympics. Finally, we close by asserting that the current formulation of the Olympics are not ‘the best we can do.' Instead, through the counterhegemonic potential of critical approaches and engaged, strategic action, a transformation of critical consciousness - and the Olympics, into something to be proud of - remain a live and entirely possible option.

## Introduction

Throughout the twenty-first century, Olympic hosts have used the Games to generate a temporary period of exceptional circumstances, using the distractions afforded by the world's largest multi-sport mega event to justify acts of surveillance, securitization, and displacement. Following the work Antonio Gramsci and his intellectual descendants, we argue that the process by which such intrusions upon civil liberties are normalized, reproduced, and contested are best theorized and understood via the Italian Marxist's reconceptualization of hegemony. In order to grasp the global implications of the Olympic industry's infringement of civil rights, as well as to identify their partners world over, this paper is also situated within the field of International Relations (IR). In particular, we build upon the foundations laid by Neo-Gramscian scholars such as Cox (1981, 1987), Gill (1993), and Worth (2011) to extract aspects of Gramsci's work from more nationally oriented topics—which guided much of his famous *Prison Notebooks*—for use on an international scale. Yet, at the same time, we embrace Worth's (2011) call to experiment with a plurality of Gramscian scholarship in IR. Here, we use an expansive, cultural approach, rooted in the works of cultural studies pioneers Williams (1973) and Stuart Hall (1983, 1986, 1990), as well as their application to sport *via* Gruneau (1999) and Hargreaves (1986).

Williams' (1973) conceptualization of cultural hegemony illustrates how the international or global interacts with the national and local in the social and cultural spheres of experience. Following Gramsci, Williams emphasized that hegemony is more than mere domination. Rather, it encompasses not only the “expression of the interests of a ruling class” but also the acceptance of these interests as “‘common sense' by those in practice subordinated to it” (p. 118). In other words, hegemony is a grand social negotiation through which a dominant group's cultural, political, and economic ideas and practices become relatively normalized. Hegemony is also a process, not some static and unchanging *status quo*, maintained by force. As Williams (1973) explains, “no dominant society or order of society, and therefore no dominant culture, in reality exhausts human practice, human energy, human intention” (p. 12). Indeed, Williams identifies resistance within the process of hegemony *via* his theorization of *alternative* and *oppositional* cultural forms. Certainly, in a democratic society, there will be “alternative meanings and values, the alternative opinions and attitudes, even some alternative senses of the world, which can be accommodated and tolerated within a particular effective and dominant culture” (Williams, 1973, p. 10). At the same time, however, there will always be *oppositional* practices, values, and ideas that are labeled grave a threat to the dominant order and “extirpated with extraordinary vigor” (p. 12).

Although in some ways theoretically dissimilar, we also consider this paper a contribution to political scientist Boykoff's (2013, 2014, 2016), previous work on “Celebration Capitalism” and the Olympics. At the crux of our argument is the symbiotic relationship between the IOC, organizing committees, and host governments, convincing the citizens of host cities that the Olympic Games are intrinsically good. It's a clear example of what Stuart Hall refers to as the “struggle for meaning” (1990, p. 77). Indeed, for the Olympics to serve as celebration capitalism, those benefiting most from the dominant, hegemonic definition of the Olympics as peaceful, progressive, and necessary, restrict access to the very language, and outlets for that language, through which opposition could be mounted. Following in the academic footsteps of Hoberman (1986, 2008); Lenskyj (2000, 2008, 2020), and Boykoff (2013, 2016), we intend to challenge that assumption by highlighting the multiple, reoccurring human right violations associated with the Olympic Games, followed by proposals to improve and/or replace the event going forward.

## Sport and International Relations: State of the Field

IR has been relatively slow to embrace sport as a topic worthy of inquiry. Writing in 1986, IR scholar Taylor (1986) lamented that the relationship, or lack thereof, between academic work in sport and IR was “a case of mutual neglect” (p. 27). As Allison and Monnington (2005) explain, little progress was made over the next two decades, concluding “the sporting dimension of IR still often plays no part in education on the subject” (p. 5). There have been exceptions to the rule. Arnaud and Riordan's (1998) edited collection *Sport and International Politics: Impact of Fascism and Communism on Sport* tackles IR in a historical context, including several important chapters on fascism (Aja, 1998; Guttmann, 1998; Krüger, 1998; Teja, 1998). Works like that of Arnaud and Riordan (1998), relating to IR, but devoid of IR scholars, or even Political Scientists more broadly, were the norm in the 1990's. As Budd and Levermore (2004) explain in their collection, entitled *Sport and IR*, studies of “sport and the international environment have been overwhelmingly written by specialists in history, law, sport studies and particularly sociology. Very little text has been devoted to sport from the academic discipline of IR” (p. 6). While both Budd and Levermore arrived at the topic from IR backgrounds, the roster of their book reflects the struggle to attract attention from IR and Political Scientists. Indeed, of the six other contributors, Lee (2004) was the only other Political Scientist. For the remainder of the twenty-first century, the observations of Budd and Levermore have largely held true.

In what we can broadly label sport studies, encompassing aspects of history, sociology, philosophy, and management, several scholars have recently drawn upon IR to push their respective fields in new directions. Jackson and Haigh (2008), for example, guest edited an issue of *Sport in Society*, gathering a diverse range of scholars and scholarship on global issues in sport. Although most of the articles came from sport studies, like historian Kidd (2008) on sport for development and peace, sociologists Maguire (2008) and Donnelly (2008), on globalization and human rights, respectively. From IR and Political Science, there were just two contributors out of a roster of twelve, including Cornelissen (2008) on foreign policy, sport, and Apartheid South Africa and Black (2008) on second-tier mega events and globalization. Scarlett Cornelissen has since become a leading scholar at the intersection of sport, politics, and IR (Cornelissen, 2010, 2011, 2012; Cornelissen et al., 2011), publishing a series of articles about the political dimensions of the 2010 FIFA World Cup in South Africa.

In the sub-discipline of International Development Studies, Darnell and Black (2011) guest edited a special issue of Third *World Quarterly* on “Mainstreaming Sport into International Development Studies,” noting that much of academia continues to view sport as “external and extraneous to serious discussions of global politics, economics and foreign policy, despite a host of analyses to the contrary” (p. 371). Here, again, we see a mix of disciplines taking sport seriously as a topic within IR. International Development scholar Robert Huish contributes a paper on Cuba, sport, and internationalism in the Global South (Huish, 2011). Particularly important for the work at hand, Political Scientist Peacock (2011) examined how the IOC's ability to adapt to “world-cultural preferences allows it to survive and have a measure of power” (p. 477). Darnell and Black's special issue of Third *World Quarterly* also illustrates the growth of “sport for development and peace” research, which has arguably become the primary topic of discussion at the nexus of sport and IR (Cornelissen, 2011; Donnelly et al., 2011; Hayhurst, 2011; Kidd, 2011; Tiessen, 2011). Much of this, however, continues to flow from historians, sociologists, and others within the sport studies sphere. Indeed, as Grix and Houlihan (2014) explain, the “relative lack of analysis of sport by political scientists and IR scholars is surprising given that sport as a political resource has a long history both externally in inter-state relations, and internally, inter alia, as part of an attempt to create a sense of statehood among citizens” (p. 574). Grix, himself, has done much to encourage increased engagement from IR and Political Science, becoming one of the few scholars to author or co-author studies of sport in mainstream politics journals, including *British Politics* (Grix, 2010), *Public Administration* (Goodwin and Grix, 2011), *Public Policy and Administration* (Grix and Phillpots, 2011), *Political Studies Review* (Grix, 2013), *Global Society: Journal of Interdisciplinary IR* (Grix and Lee, 2013), *British Journal of Politics & IR* (Grix and Houlihan, 2014), amongst others.

Only a handful of scholars have specifically turned their attention to the place of the IOC and Olympic Games in the context of IR. Political Scientist Boykoff (2016), whose work we discuss in more detail later, has provided the most up-to-date political history the Olympics, building on previous monographs by Hill (1996) and Espy (1979). Peacock, in addition to his work noted above, has provided important contributions on the IOC's place in the United Nations (Peacock, 2010); territorial disputes played out in the Olympic context (Peacock, 2008); and Olympic imperialism (Peacock, 2006). Beacom's (2012) monograph *International Diplomacy and the Olympic Movement* provides a useful overview of everything from bids to boycotts against the backdrop of IR or, more specifically, what Cox (1983, 1987) theorizes as “world order.”

## Manufacturing Consent: IOC Hegemony, World Order, Local Consequences

In his prison notebooks, Italian Marxist Antonio Gramsci teased out the process of hegemony, illustrating how a single social group can exert its influence over a nation by dominating certain civil institutions, like schools, churches, and media, thereby shaping society in its image (Gramsci, 1978). Although we view our critique of the Olympics and human rights as part of the broader Marxist tradition espoused by Brohm (1978) and Perelman (2012), who primarily mobilized the work and style of the Frankfurt School, we stop short of writing the sort of polemical treatises that characterize this tradition, in favor of a more stayed and analytical approach shaped by Gramsci. We concur with Gruneau's (1999) reading of Brohm, viewing his “arguments are powerful, penetrating, and greatly overstated,” emerging from a “polemical tradition of revolutionary neo-Marxism,” which nonetheless represent a “welcome alternative to the numerous uncritical celebrations of sport that have passed for social scientific, historical, and philosophical analyses…” (p. 15). It is important to emphasize, however, that we are nonetheless inspired and influenced by the likes of Brohm and Perelman, as well as the Frankfurt School scholars who came before them, like Horkheimer and Adorno. Indeed, we concur with Horkheimer's (2002) general view that critical theory should pursue the “the abolition of social injustice” (p. 242). Furthermore, Brohm provides an important jumping off point for our theoretical orientation, framing sport as an institution of indoctrination, representing “one of those secondary arms of the state” capable of imparting national practices, meanings, and values (p. 55).

This article tackles a gap in studies of hegemony and sport, and hegemony and cultural forms more broadly, by bringing together the uses (and some cases abuses) of hegemony in cultural studies and IR, to tackle the international sporting landscape. In many ways, we consider our work as a continuation of the Coxian school of IR, in that our analysis of hegemony at the global level relies heavily on our reading of Cox (1983, 1987) and his application of Gramsci to world order. As Cox (1983) explains:

Hegemony at the international level is…not merely an order among States. It is an order within a world economy with a dominant mode of production which penetrates into all countries and links into other subordinate modes of production. It is also a complex of international social relationships which connect the social classes of the different countries (p. 171).

Yet, as Worth has shown, there remains much opportunity within the realm of Coxian IR to experiment with hegemonic scholarship from the broader social sciences, most notably the cultural studies of Williams and Hall. Worth (2015) himself makes such an effort in his underappreciated monograph *Rethinking Hegemony*. Sport, however, does not factor into Worth's ambitious analysis of recent world events. While Brohm (1978) employs Gramscian hegemony to sport and the national, he has little to say regarding hegemony and the international or global. Though Gruneau makes far more use of Gramsci, as well as Williams and Hall, his focus is primarily on sport within Canada. Hargreaves (1986) performs a similar analysis of Britain, addressing the rise of international sport while stopping short of examining it as a hegemonic, cultural form within a world order.

The IOC and the Olympics benefited from incorporation into two successive world orders. During the first, which Cox (1987) calls “the era of rival imperialisms” (p. 109), the Olympics enjoyed the patronage of multiple colonial regimes, the largest and most influential of which was the British Empire. This gave way to the “neoliberal world order” following World War II, marked by increased American influence.^1^ The origin and rise of neoliberalism are a matter of debate, but important watershed moments can be identified. First, after World War II, the United States of America took a dominant position in global hegemony, primarily through its leading role in the foundation and maitence of Bretton Woods institutions in the 1940's, including the International Monetary Fund (IMF) and World Bank, as well as the fledgling United Nations (Harvey, 2005).

Much of what Pierre de Coubertin based the modern Olympics on was derived from the sporting cultures of English public schools and American universities. Coubertin celebrated the athletic traditions of Ancient Greeks and undoubtedly drew some inspiration from the physical cultures of Sweden and Germany, but it was the British and Americans he and his Olympics owed the greatest debt to. Still an Empire at the time of the first modern Olympics in 1896, the British spread their fondness for Olympism and Coubertin's efforts across the globe via their network of overseas possessions, quickly drawing competitors to the Games from the settler-regimes of Canada, New Zealand, South Africa, Australia, and India. It is not enough for governments to believe in the IOC's mission. Indeed, the people of these nations must deem the mega event necessary and desirable for competition or hosting to remain feasible.

During the era of rival imperialisms—a time when Britain's global political, cultural, and economic influence generally prevailed—governments with British Imperial roots like Canada, Australia, New Zealand, and South Africa all entered the Olympic movement, at once celebrating their Britishness and constructing their uniqueness within the Empire. The United States of America (USA), which broke with the British Empire via violent revolution, could compete against its old foe in the sporting arena, generating much enthusiasm amongst its citizenry for competing in and following the Olympic Games. After WWII, America's global influence surpassed that of the British, marking the beginning of a new, neoliberal world order. This American-led world order has meant, above all else, the aggressive ascension of capitalism over all other economic models and globalization of neoliberalism via international institutions like the World Bank and International Monetary Fund (Cox, 1983). The IOC's ability to shift and welcome new types of elites, thus maintaining its important social connections within the world order is always evident. As Brohm (2009) explains, the IOC “develops an expansionist diplomacy intended to expand the Olympic empire in a double game of commercial seduction and political allegiance to international institutions commercial seduction and political allegiance to international institutions (UN, G8, UNESCO, etc.), donors (IMF, World Bank) and major powers, especially emerging nations or what is now called the “BRIC” (Brazil, Russia, India, China) (p. 79).^2^

Under such conditions, sport—and particularly Olympic sport—became politically charged. A line was drawn, with the capitalist USA and its allies on one side and the Communist Soviet Union and socialist republics on the other. As early as 1933, the Soviet Union was officially evaluating the value of athletic dominance over their capitalist counterparts (Allison and Tomlinson, 2017). The sportive struggles between the USA and USSR, or the “sporting arms race” as they have been dubbed, are well documented by historians and need not be rehashed here (Torres and Dyreson, 2005; Rider, 2016, 2018, 2019; Congelio, 2018; Hunt, 2018; Ross and Nagel, 2018). With American millionaire Brundage at the helm between 1952 and 1972, the Olympics were firmly within the USA's sphere of influence. In short order, Olympic “practices, meanings, and values” (Williams, 1973) increasingly reflected Brundage's America and the world order it constructed. As communications scholar Marvin (1981) suggests, Brundage viewed Olympic sport as a vehicle for Americanization, “essential for the continued success of American capitalism at home and abroad” (p. 81). Yet, counter intuitively, he saw no place of commercialism within Olympism or vice versa (Wenn and Barney, 2000). The autocratic Brundage was a staunch supporter of strict amateurism—devoid of any sponsorship and financial enticements of any kind—and an unabashed elitist at heart. The Soviet Union had subverted amateurism to communism, permitting the sort of mass participation and compensation that Brundage found so distasteful. He was a “proponent of administrative patronage by the rich and the privileged,” leaving the athletes to toil at the whim of the world's elites (Tomlinson, 2012, p. 235). Yet, his affection for American capitalism, and all the inequality that came with it, never spilled over into an unreserved embrace of the commercialization of the Olympic Games.

While Brundage's elitist view of the Olympics resulted in a hesitancy toward the televising of events, Juan Antonio Samaranch came into the role of IOC President with a more business-orientated outlook. Under Samaranch, commercialization was not just embraced, it was prioritized. As the Olympics limped through the 1970's, with the terrorist attack at the 1972 Munich Olympics and massive debts of the 1976 Montreal Olympics generating global antipathy toward hosting, Peter Ueberroth sought to reconstruct hosting along thoroughly neo-liberal lines (Wenn, 2015). As chairman of the Los Angeles Olympic Organizing Committee, Ueberroth and his team primarily secured private funding for the 1984 Olympics, transforming the Games into the massive, corporate spectacle we know today (Boykoff, 2016). Over the years, Ueberroth's model for privately-funded Olympic Games (albeit with plenty of government support) mutated into what political scientist Boykoff (2013, 2016) has dubbed celebration capitalism.

## Hegemony and Celebration Capitalism

In his *Power Games: A Political History of the Olympics*, political scientist Boykoff (2016) describes celebration capitalism as “a political-economic formation marked by lopsided public-private partnerships that favor private entities while dumping risk on the taxpayer. The normal rules of politics are temporarily suspended in the name of media-trumpeted, hyper-commercial spectacle, all safeguarded by beefed-up security forces responsible for preventing terrorism, corralling political dissent, and protecting the festivities” (p. 155). Inspired by the works of Agamben (2005) and Klein (2007), Boykoff's celebration capitalism neatly encompasses elements of each, representing a powerful and underappreciated view of Olympic hosting. In Shock Doctrine (Klein, 2007), Klein rallied against disaster capitalism, as expounded by economists Friedman and Friedman (1982) in the preface to *Capitalism and Freedom*:

“There is enormous inertia—a tyranny of the status quo—in private and especially governmental arrangements. Only a crisis—actual or perceived—produces real change. When that crisis occurs, the actions that are taken depend on the ideas that are lying around. That, I believe, is our basic function: to develop alternatives to existing policies, to keep them alive and available until the politically impossible becomes politically inevitable” (p. vii)

Klein observed a Machiavellian thread in Friedman and Friedman's vulture-like disaster capitalism, which seizes upon distraction and discord to inflict political and economic wounds upon the masses, startlingly reminiscent of the surreptitious maneuvering advised in *The Prince*: “Injuries, therefore, should be inflicted all at the same time, for the less they are tasted, the less they offend” (Machiavelli, 1998, p. 33). The injuries described by Machiavelli occur in the vacuum of exceptional circumstances, swiftly perpetrated upon the conquered, but with consequences that reverberate far into the future. In his book *State of Exception*, philosopher Agamben (2005) argues that political leaders routinely leverage exceptional moments of disaster and crisis to introduce measures that restrict freedoms, exploiting a distracted populace in their time of need. Boykoff (2013, 2016) convincingly argues that governments employ the same repressive techniques during large-scale festivals, including the Olympics and Paralympics, using collective jubilation as a backdoor to oppression.

While the IOC's influence is most pronounced in host cities, states, and nations, where they generate the kind of exceptional circumstances outlined by Boykoff (2013, 2016), all host cities, to varying degrees, incorporate Olympic “practices, meanings, and values,” which, according to Williams are diffused to the wider population via the schools, media, legislators, and official government statements, entering a collective—but not universal—common sense (1973). As Boykoff (2016) explains, the IOC nurtures a state of social euphoria through various binding documents, requiring the OGOC and NOC to pursue “the fullest possible broadcast and other media coverage of the Games and the widest possible audience for the Games” (International Olympic Committee, 2017, p. 8). Typically, when the media egregiously ignores the very real social consequences of some major corporate development, protests follow close behind. The IOC does its utmost to repress such freedom of expression, speech, and assembly, opposing anything that may limit the social euphoria of the games. This government intervention, despite public affirmations of neo-liberalism, is not surprising given the state of global capitalism. As Robinson (2014) observes “Capitalist globalization is an ongoing, unfinished, and open-ended process, one that is contradictory and conflict-ridden, driven by social forces in struggle” (p. 2).

Cox (1983) theorizes a grander, global hegemony, through which “historically, a state would have to found and protect a world order which was universal in conception, i.e., not an order in which one state directly exploits others but an order in which most states (or at least those within reach of the hegemony) could find compatible with their interests” (p. 171). The international consent for such a hegemony is primarily obtained via international organizations, such as the United Nations, International Monetary Fund, World Bank, and/or IOC. We argue that the IOC, as the dominant body in global sport, encompassing 206 National Olympic Committees (NOCs), serves to facilitate “the expansion of the dominant economic and social forces” of the most influential State within the global hegemony, while at the same time permitting “subordinated interests with a minimum of pain” (p. 172). As a non-governmental organization, however, the IOC is rather more politically vulnerable than its inter-governmental counterparts. To reinforce its status as a global sporting leader, therefore, the IOC sought out allies within the dominant, hegemonic segments of the global political system, particularly the United Nations (UN) and World Health Organization (WHO).

“The existence of the possibility of opposition, and of its articulation, its degree of openness, and so on,” writes Williams (1973), “again depends on very precise social and political forces” (p. 10). Leading up to any Olympic Games, the “social and political forces” prevailing in the host city and nation become entangled with the goals of the IOC, enshrined in the Olympic Charter and Host City Contract. It is a symbiotic relationship between the IOC and the host city, state, and nation. Regardless how pervasive and persuasive a dominant, hegemonic way of thinking becomes, the possibility of resistance always remains. In many nations, some degree of resistance is even celebrated. Indeed, peaceful protests and demonstrations for civil liberties and human rights are permitted within the machinations of liberal democracy. After all, many such governments claim a degree of solidarity with protestors against racism, sexism, homelessness, and so on. These sorts of social issues can easily be incorporated into a dominant, hegemonic culture, whereby the political apparatus issues platitudes and minor concessions to douse the flames of resistance and, indeed, claim some ownership of said resistance.

When the IOC and host act as one, groups engaged in resistance are no longer viewed as merely “disregarding or despising” the status quo, but actively “challenging it” (Williams, 1973). Section 50.2 of the *Olympic Charter* reads: “No kind of demonstration or political, religious or racial propaganda is permitted in any Olympic sites, venues or other areas” (IOC, p. 90). Under the guise of a state of exception (Agamben, 2005), government officials appease the IOC, extinguishing the freedoms of speech, expression, and assembly for the duration of the games. Indeed, acts of resistance that would typically be viewed as the expression of alternative ideas or values are reframed and identified as oppositional. That is, they are *officially* labeled a threat to the Olympic Games and, given the immense financial investments made to organize such mega events, the image and budget of the host. How to quash such freedoms is left to the host, with input from the IOC, resulting in the normalization of legislation infringing upon the civil liberties of the host city's population in various ways, including the expansion of police powers, displacement or imprisonment of “problematic” groups, and implementation of new security and surveillance technologies. In most cases, at least part of the security apparatus developed for the Olympic Games is repackaged and directed toward citizens of the post-Olympic host city. In this way, the IOC has a lasting, negative impact on civil liberties in host cities. Municipal and state authorities are complicit in the oppression. In the following analysis, we examine how these repressive techniques have grown and evolved in the twenty-first century, from 2000 Sydney Olympics to the 2016 Rio Olympics.

## A Promising Start? The 2000 Sydney Olympics and the Illusion of the “Best Olympics Ever”

During the 1980's, Australian politicians became increasingly interested in globalization and what it could mean for the nation. It was a time of significant reorientation in the World Order. The collapse of the USSR ushered in an era of American, neoliberal influence on a global stage, free and clear of its old communist ideological rivals. As Worth (2015) explains, Ronald Reagan and Margret Thatcher laid the ground work for this global market shift within the USA and United Kingdom, respectively. The influence of Reagan, in particular, should not be underestimated. Indeed, as Cox (1987) suggests in *Production, Power, and World Order*—the seminal text on global hegemony—“a world hegemonic order can be founded only by a country in which social hegemony has been or is being achieved” (p. 149). From Reagan onward, neoliberalism became America's economic “common sense,” continuing under Republican and Democratic presidents, alike. American influence at the World Bank and International Monetary Fund assured that this national neoliberalism could emanate beyond state borders, becoming a global economic rational, initially guided by North Americans and Europeans, before ultimately replicating elsewhere, mutating into a transnational capitalist class (Worth, 2015). Australian Prime Minister Paul Keating was determined to use the Olympics to further integrate his nation into the neo-liberal world order. Beijing had similar aspirations and put forward a strong bid of their own. Rather than risk losing a narrow vote, Australian Olympic Committee (AOC) President John Coates made donations of $35,000 USD to sports programs in both Uganda and Kenya, stating “it might encourage them to consider their votes for Sydney” (Coates, 2011). It did. The IOC awarded the 2000 Summer Olympics to Sydney by a margin of exactly two votes.

For Keating, securing the Olympic Games was about more than just hosting an extravagant global mega event. It was about bringing some of the world's biggest brands and employers to Australia. Eight months after the Games were awarded Keating emphasized that “globalization, competitiveness and productivity” were central to his vision of Australia's future (Keating, 1994, p. 56). As Toohey (2008) explains, there was hope that “the media and business interest generated by the games would improve Australia's international profile as a safe, stable and financially secure economy for other trading nations” (p. 1,960). Keating did not get to see the Olympic project through to completion. He was toppled from power by John Howard's coalition government in 1996. Although Keating was certainly taking Australia down the neo-liberal path, Howard expedited and expanded such efforts. As Greenfield and Williams (2003) observed, Howard's lengthy tenure as Prime Minister was marked by “constant repetition of individualist corporate rhetoric and policy settings, and their battering of people with both national chauvinism and, at the same time, corporate-led globalization” (p. 294).

Australian officials did their utmost to craft a narrative of inclusion, harmony, and peace around the 2000 Sydney Olympics. On 25 November 1997, Australian Representative Penelope Wensley addressed the United Nations (UN) General Assembly in New York City regarding the upcoming 2000 Sydney Olympic Games. According to Wensley, the presence of the UN flag at the Games would “be a visible daily reminder of the shared ideals of the United Nations and of the International Olympic Committee, something that will reaffirm visibly, simply and directly the importance of the United Nations and the commitment of all participants in the Olympics not just to sport and the ideal of sporting prowess, but to the promotion of international cooperation” (p. 11). The UN's human rights functions, however, soon derailed much of the optimism surrounding the 2000 Sydney Olympics. Australia was and is party to the *International Convention on the Elimination of All Forms of Racial Discrimination* (CERD). As Christine O'Bonsawin (2015) had shown, the Howard Government's 1998 revisions of the *1993 Native Title Amendment Act* eliminated many of the land rights enjoyed by Indigenous communities, much to the benefit of the settlers and corporations. The UN ruled that the Australia was indeed in contravention of its CERD obligations and censured the nation to that effect (Robbins, 2007). Howard's eagerness to trim the land rights of Indigenous communities was directly tied to his commitment to supporting and expanding the nation's extractive industries. Upholding the power and potential of corporations, in Howard's eyes, was more important than respecting the original inhabitants of Australia.

Writing on hegemony, Hall (1988) underlined the power of the State—Howard Government or otherwise—to regulate discourse:

The State is frequently the primary agency through which cultural relations are organized and reorganized. One need only think of relations between the ideological fields of public or popular opinion and the institutions of civil society—newspapers, the mass media, educational institutions, and the church—in terms of access both to the technology and the means of the formation of people's identities, to realize that it the State which regulates many of the forms in which cultural and ideological production take place (p. 163).

The Howard Government was quick to attack institutions it felt were contradictory to its neoliberal aims and interpretation of Australian culture. To sabotage left-wing intellectuals, who regularly illustrate the immense inequities driven by the neoliberal World Order, the Howard Government slashed public funding for universities and interfered in the Australian Research Council, sabotaging grant proposals inconsistent with its view of Australian society. The Australian Broadcasting Company (ABC) not only saw its budget reduced by $55 million ASD, but its staff replaced with right-wing loyalists like Janet Albrechtsen of News Limited to the broadcaster's board (Bonnell and Crotty, 2008). Howard also carefully nurtured an assault on political correctness in politics, elevating the place of racist rhetoric in Australian society. When Pauline Hanson, founder of the far-right One Nation Party, delivered a racist assessment of Australia's future, Howard refused to denounce her. Howard also refused to apologize to Indigenous communities following the Stolen Children Report, which highlighted the Australian Government's practice of removing “children of mixed parentage from their Aboriginal parents” (Hocking and Stern, 1998, p. 412).

The Labor Government of Bob Carr in New South Wales also garnered unwelcomed attention during Olympic preparations, ushering in sweeping changes to legislation in New South Wales, reigning in civil liberties in the name of the Olympics. Instead of an era prioritizing human rights, bringing the Olympics in line with UN ideals, the 2000 Sydney Games proved a turning point in the securitization, surveillance, and ongoing violation of civil liberties by host cities long after the closing ceremonies. Although this can, in part, be attributed to a successful domestic terrorist attack at the 1996 Atlanta Olympics, whereby an anti-abortionist detonated a bomb at the Centennial Olympic Park, killing one person and wounding 111 others (Yarborough, 2002; Jennings, 2012; Boykoff, 2016), the long-term consequences of surveillance, securitization, and displacement sparked shockingly little concern from Olympic organizers.

The key to such intrusions on civil liberties lays in convincing the majority of a host city that such measures are momentary inconveniences, necessary to host the Olympics, but not indicative of what day-to-day affairs will be like in the future. Organizers seize upon traditional Olympic rhetoric to sell the Games as inherently good and worthy of sacrifice. The Olympic values becomes the hosts' values. In Australia, the Olympics, and by extension the IOC, were incorporated into the dominant, social and cultural norms of Australian society years before the opening ceremonies by leveraging institutions of indoctrination. According to Williams (1973), “The educational institutions are usually the main agencies of the transmission of an effective dominant culture, and this is now a major economic as well as cultural activity; they are both in the same moment” (p. 9). Sydney won the right to host the 2000 Olympics in 1993. By 1995, the ASPIRE^3^ Program was underway, taking Olympic education into the nation's schools, to teach “young Australians on the values, spirit and philosophy of the Olympic Movement” (IOC, n.d.). When the Olympics rolled around 5 years later, those same children were now young adults, predisposed to see the Games in a positive light. For the adult population, newspapers were signed on as official Olympic outlets. Politicians employed the rhetoric of reconciliation to make settler Australians feel better about their colonial past, while doing little to actually improve the well-being of the island continent's original inhabitants. All of this washed over the Australian public, repeated over and over again *via* the media, fusing with the nation's dominant set of “meanings and values” (Williams, 1973, p. 9).

While Australians' senses were bombarded with positive messages about the Olympics, caught up in the euphoria of the moment, organizers and politicians used the distraction to fulfill their own Olympic dreams. As historian Lenskyj (2002) has shown, the Public Interest Advocacy Center (PIAC) raised concerns about the impact of Olympic-related securitization on the people of Sydney, particularly “the poor and homeless, sex trade workers, people with disabilities, and sexual and racial/ethnic minorities” (p. 51). Yet, by focusing overwhelmingly on the safety of tourists, competitors, and other Olympic visitors, government and organizers largely sacrificed the liberties of their most vulnerable residents. Furthermore, the new legislation was rather fluid and open to interpretation, placing limited restrictions on the suppression of civil liberties. The *Police and Public Safety Act*, for example, granted police near unlimited power to stop and search any individual they thought might be concealing a weapon. Police only required “reasonable” grounds to conduct such searches, which included *suspects* simply being “in a location with a high incidence of violent crime” (p. 52). As a result, explains Australian law professor Head (2000), the police enjoyed “vague and wide-ranging powers, subject to minimal safeguards for civil liberties” (p. 132).

As the Olympics grew nearer, both the Federal and New South Wales governments ramped up their security measures. Under what Head (2000) labels “Olympic Security Legislation”—encompassing a series of New South Wales laws including the *Homebush Bay Operations Act 1999 (HBOA), Security Industry (Olympic and Paralympic Games) Act 1999, Sydney Harbor Foreshore Authority Regulation 1999, Olympic Arrangements Act 2000—*civil liberties were further depleted, removing even “minimal procedural safeguards” (p. 132). Individuals deemed “enforcement officers” were granted powers above and beyond their counterparts in the police. They didn't need to identify themselves to the accused. They could remove people without warning and ban them from Olympic sites. They could search people and their belongings and seize property (Head, 2000). Not all of these measures were temporary. When the *HBOA* was about to expire, the NSW government simply replaced it with the *Sydney Olympic Park Authority Act 2001*, reviving and extending the *HBOA*'s limits on freedom of expression (Legal Observer Report, 2002).

Although there was no forced displacement at the 2000 Sydney Olympics *per se*, the outcome for many low-income renters was the same. The rapid gentrification of areas near the Olympic venues sparked large-scale renovations, often resulting in evictions and displacement. An NGO called Rentwatchers, assisted by the Green Party, introduced legislation to limit rent increases and evictions for the duration of the Olympic Games, only to see it struck down by the New South Wales Parliament (Centre on Housing Rights Evictions, 2007). Without clear, quantitative data linking gentrification to the Olympic Games, the government refused to intervene. Although it was private landlords who perpetrated the evictions, rather than State or City officials, the outcome was the same: displacement. After all, why would officials intervene? The landlords did the dirty work of displacement for them, beautifying and upscaling their buildings, hanging the window dressing of progress and stability for federal, state, and municipal governments, all anxiously preparing for the eyes of the world. The IOC served as a legitimizing force in this gentrification, giving politicians and citizens the justification they needed to simply ignore or dismiss the Green Party and Rentwatchers, resting easy in the assumption that short-term sacrifices in the name of Olympics would somehow benefit the people of Australia well into the future.

As Toohey and Taylor (2012) demonstrate, a broadening of police powers and expansion of surveillance technologies remained in Sydney after the Olympics, providing Sydney with a legacy of “increased government intrusion into civil liberties” (p. 335). Beginning in 2000, such measures, and how to construct them, were documented and passed on from host to host *via* the Olympic Games Knowledge Management Programme (OKMP). Indeed the OKMP encourages, and provides a blueprint for, the securitization of host populations via security legacies. In hegemonic terms, the IOC leverages the OKMP to simplify and normalize securitization and displacement for OGOCs. For host nations, eager to reinforce their own mechanisms of dominance, the Olympic and Paralympic Games serve as a Trojan Horse of sorts, ushering in long-term infringements of civil liberties under the guise of a momentary and necessary exception to the status quo.^4^ Yet, since at least the 2000 Sydney Olympic and Paralympic Games, what are touted as transient measures, necessary for the duration of the Games, are seized by State actors and used against their own citizens long after the closing ceremonies. The 2000 Sydney Olympics, despite the *HBOA* and displacement of citizens via gentrification, were the least oppressive Summer Games of the twenty-first century. Unfortunately, the initiation and implementation of the OKMP for the 2000 Sydney Olympics made strategies for the violation of civil liberties something that could be packaged and sold, resulting into the distribution of these methods to the 2004 Athens Olympics and every Games since. It does not need to be this way. The IOC has real emancipatory potential.

## The Post-9/11 Realities of Olympic Hosting and Long-Term Securitization

On 11 September 2001 (9/11), the terrorist group Al-Qaeda flew two jet liners into the World Trade Center in New York City. A staggering 2,977 victims lost their lives, constituting the deadliest terrorist attack ever recorded. The task of organizing the first Olympics of the post-9/11 era fell to the Athens 2004 Organizing Committee (ATHOC). As the home of the ancient Olympic Games and the first modern Olympic Games, Greece's dominant cultural discourses were already steeped in Olympism long before Athens won the right to host in 1997. Indeed, Greek Olympic education efforts started in 1961, following the completion of the International Olympic Academy. These efforts increased leading up to the 2000 Athens Olympics, more thoroughly incorporating Olympism into the primary and secondary school curriculum (Makris and Georgiadis, 2017). Ironically, a major thrust of the Olympic pedagogy was developing “a critical approach to contemporary problems and sports-related issues” (Makris and Georgiadis, 2017, p. 48). Although these so-called Olympic “meanings and values” (Williams, 1973, p. 9) were vigorously incorporated into the dominant Greek culture of the time, the Greek and Athenian governments and ATHOC were preparing to implement a number of measures that would promptly become contemporary problems of their own. Indeed, while Greek children and youth were told to think critically and embrace humanitarian goals, celebrating the lofty ideals of good will and selflessness, organizers used this moment of exception to install new technologies of mass surveillance and displace vulnerable communities, often inhabited by the Roma minority, to clear the way for the Olympic Games.

The ATHOC's security spending dwarfed that of the SOCOG. While security for Sydney amounted to $179.6 million, Athens' spending soared to a whopping $1.5 billion, an increase of roughly 831% (Boyle and Haggerty, 2009). Although it was the ATHOC that was ultimately responsible for security measures for the 2004 Athens Olympics, there was significant international involvement in the process, spearheaded by a seven nation Olympics Advisory Security Team (OAST), consisting of America, Germany, France, Britain, Australia, Israel, and Spain (Samatas, 2007). Realistically, the ATHOC had little choice but to accept the “help” of their international allies. As Samatas (2011) explains, “Greece was required to build this international security alliance and obliged to purchase the latest US and EU security and surveillance technology in order to obtain international support and confidence, and thus to avoid boycotts and cancellation of the Games” (p. 3,351). Greek officials viewed such collaboration as an opportunity to bolster the securitization of Greek society over the long haul and become leaders on the global security market. Indeed, leading up to the Games, Floridis (2004), a former Greek Minister of Public Order, observed:

Large sums of money, indeed, have been invested in the planning of security for these games. This great expenditure, however, is not concerned only with the duration of the Olympics. It is an investment for the future. The special training, technical know-how, and ultramodern equipment will turn the Hellenic Police into one of the best and most professional in the world, for the benefit of the Greek people (p. 4).

Although Greek security professionals were indeed consulted by the Beijing Organizing Committee for the Olympic Games (BOCOG), the nation's newfound security advancements were more commonly focused upon its own population. As the Olympics ended and venues fell into disuse, the surveillance web supposedly put into force to protect the Greek people quickly became a weapon of the state, resulting in the “permanent introduction of extensive surveillance devices and the ensuing contraction of civil rights and liberties” (Tsoukala, 2015, p. 294).

In addition to leveraging the Olympic Games for the normalization of widespread surveillance, the Greek government also used the Games to justify increased marginalization of the country's Roma minority. It was not the first time that the Roma received such treatment at the hands of Greek authorities. In 1983, as Law et al. (2014) explain, “the Greek government sanctioned the racial segregation and ghettoization of the Roma in a ministerial decree, justifying this as ‘cleaning operations' and constructing the Roma as social dirt” (p. 112). Although the 2004 Athens Olympics included infrastructural and housing improvements that benefitted many citizens, Roma minorities (~2,700) were unsurprisingly and disproportionately targeted for forced eviction (Centre on Housing Rights Evictions, 2007). For Roma, the Athens Olympics exacerbated historical marginalization, discrimination and the large-scale and aggressive forced evictions that they have been subject to, and which have seen Greece condemned by regional and international human rights organizations (Centre on Housing Rights Evictions, 2007). In 2004, Greece was found to be in violation of the *European Social Charter* through the systemic denial of adequate housing to Roma. Both the Greek National Commission on Human Rights and the UNCESCR, (in their initial report of Greece under ICESCR) concluded that the Olympics were used as an opportunity to unlawfully drive Roma from many regions (Centre on Housing Rights Evictions, 2007). Further, local authorities (often untruthfully) used the pretext of necessary Olympic construction to validate and hurry along the process of eviction, which were often carried out without compensation, due diligence or adherence to existing Greek legal procedures for eviction; indicative of the systemic discrimination that Roma faced in Athens Olympic preparation (Organisation Mondiale Contre la Torture, 2004a,b; Centre on Housing Rights Evictions, 2007).

## Olympic-Backed Authoritarianism: The 2008 Beijing Olympics

On 13 July 2001, the IOC selected Beijing to host the 2008 Olympic Games by a wide margin, prompting escalated securitization by the ruling Communist Party of China (CPC). At the time of the IOC's vote, the CPC was already well-known for its human rights abuses, ranging from restrictions on the freedoms of speech, press, and expression, to more serious accusations the suppression, displacement, and torture of Tibetans. For the embattled regime, an Olympic Games was the perfect opportunity to sport wash its previous human rights infractions and portray itself as an ambassador for the IOC's brand of peaceful internationalism. In doing so, the CPC strengthened its position within the neoliberal world order, flexing its economic muscle by providing a spectacle that not even the ficklest capitalist could take issue with. As Caffery (2011) observed, the 2008 Beijing Olympics sent “a message of intent that pointed to a larger Chinese plan for reengagement with the world to a degree not seen since the early Ming Dynasty” (p. 142). It was all for political advantage within the global hegemonic order. Indeed, as former CPC General Secretary turned activist Tong (2008) explained, “no other government has been quite so eager to use the Games as a ploy to enhance its political prestige” (p. 249). The IOC was happy to help.

Leading up to the 2008 Beijing Olympics, China's position in the prevailing World Order was a matter of much debate. In the late 1970's, the CPC began shifting China's economy away from strict state socialism to a more market-influenced model, facilitating foreign investment and privatization. During the 1990's and 2000's, China's economy boomed. As urban studies scholar Wu (2008) observes, “the Chinese case shows that neoliberalization is the trajectory to establishing a market society, a direction of greater market re-orientation in the world, albeit the fact that different routes are followed in different countries” (p. 1,093). The CPC was eager to showcase the nation to the world. In 1993, Beijing bid for the 2000 Olympic Games, but lost out to Sydney, Australia. Leading up to the decision, China's human rights record was put under a spotlight, sparking widespread condemnation of the regime. Although the Chinese human rights record was indeed grim, they had reason to be frustrated with the IOC's decision. Not only had Sydney organizers clearly wined, dined, and bribed their way to hosting the 2000 Olympics, the voting membership of the IOC was two-thirds European, casting a specter of racism over the proceedings (Xu, 2008). A year after Sydney played host to the world, Beijing and China got their wish, winning the rights to host the 2008 Olympics. What was a dream come true for the CPC proved a nightmare for others.

As had been the case leading up to and during the voting for the 2000 Olympics, the CPC's poor human rights record sparked ridicule from nations prior to the 2008 Games, but Olympic leaders were confident that such fears were unfounded. In fact, according to the IOC, the Olympics would help bring China in line with international human rights norms. IOC President Jacques Rouge, for example, hoped that Beijing serving as an Olympic host would “do a lot for the improvement of human rights and social relations in China” [as quoted by Kim (2006)]. When Amnesty International (AI) and Human Rights Watch (HRW) both issued public complaints, highlighting the intensification of CCP human rights abuses between 2001 and 2008, the IOC refused to take action (Amnesty International, 2008a,b; Human Rights Watch, 2008). At the UN, the International Federation for Human Rights (IFHR), Reporters Without Borders (RWB), and Human Rights in China (HRC) all likewise raised alarms about the abuses unfolding in the PRC. “Nobody apart from the International Olympic Committee seems to believe any longer that the government will make a significant human rights concession before the before the Games start,” stated RWB to the UN Human Rights Council (United Nations Human Rights Council, 2008). The IFHR and HRC noted that resistance to human rights violations were met with heavy-handed suppression. “Continued detentions a heavy sentences for journalists, lawyers, Internet activists and other human rights defenders reflect the Chinese government's hardening attitude in the lead up to both the 17th [Communist] Party Congress and the Beijing Olympic Games in 2008” (United Nations Human Rights Council, 2007). The IOC's desire to welcome a rising super power like the People's Republic of China (PRC) into the fold, especially after the nation's flirtation with the notion of alternative events like the Games of the New Emerging Forces, trumped any and all human rights concerns. The expansion and reinforcement of IOC hegemony is always the priority.

Although it is important to be cautious when assessing the China's human rights record and avoid what Chow (1998) has dubbed the “King Kong Syndrome,” whereby western scholars view the CCP through an Americanized lens, “as a spectacular primitive monster whose despotism necessitates the salvation of its people by outsiders” (p. 94), abuses surrounding the 2008 Olympic Games are well-documented. With the benefit of hindsight, the wide ranging human rights violations reported before, during, and after the 2008 Beijing Olympics are clear and irrefutable. Although aspects of the CPC's rule relies on persuasion, particularly thorough education and media, the threat of force—imprisonment, relocation, execution—is always present. The IOC permitted authoritarian regimes to host in the past, but each time the consequences of permitting such hosts were not fully realized until long after the Olympics left town. The obvious example was Hitler's use of the 1936 Berlin Olympics for Nazi propaganda, but the IOC failed to learn and adapt from its flirtation with the Nazis. Leading up to the 1968 Mexico City Olympic Games, ruled via single-party authoritarianism, the government massacred hundreds of students for protesting the Olympic Games at Tlateloco Square. The build up to the 1988 Seoul Olympics, awarded to Dictator Park Chung-hee, resulted in the displacement and/or internment of thousands, and the rape and murder of an untold number of Koreans as the regime prepared for the Games. The CPC's assault upon its own population, in the name of the Olympics, was completely foreseeable.

The CPC allocated a reported $21.7 billion for over 140 Olympic-related projects and spent a further $40 billion on infrastructure (e.g., transportation, energy network, water/sewage systems, and urban environment) to completely transform Beijing into the image of a livable, functional, cultural metropolis (Wang et al., 2015). Dubbed the “Grand Beijing Safeguard Sphere,” the CCP's safety and security scheme was initiated in 2001 and came to include, amongst other things, 300,000 CCTV cameras in Beijing alone, new personal identification cards, and facial recognition software (Boyle, 2012). The government's ability to track the citizens of and visitors to Beijing represented the most complex security apparatus in the world. With the so-called “Great Firewall of China,” the CCP took this surveillance to the internet, allowing the regime to scrutinize online interactions. How could such technology be developed and deployed so quickly? Like the AOTHC in Greece, the CCP and BOCOG benefited from much international assistance. Although it was technically illegal for American companies to be involved arming Chinese forces, providing security technology for industrial purposes was permitted, and that's exactly what General Electric and IBM did, supplying much of the surveillance technology employed by the CCP and BOCOG (Boykoff, 2016). Beijing officials also benefited from the OKMP and meetings with Greek security experts, well-versed in mass surveillance and leveraging the Olympics for post-Games securitization. Indeed, according to Greek sociologist Samatas (2011), “Athens 2004 delegation comprising 24 company executives visited Beijing from 31 October to 3 November 2004,” after which “a group of 39 Chinese officers was sent to Greece to learn from the Athens Olympics security model” (p. 3,351). Like the ATHOC before it, Beijing also gathered a broad, international array of security experts, representing 75 security firms from across 12 countries (Yu et al., 2009).

IOC President Jacques Rogge claimed there would be no internet censorship at the games, but journalists working in the press center found access to Amnesty International, BBC (Chinese language), Radio Free Asia, as well as “several Hong Kong newspapers known for their freewheeling political discourse” all blocked (Jacobs, 2008). The BOCOG tried to explain away the censorship as merely defective websites, but the fact that websites detailing the 1989 Tiananmen Square massacre and other human rights violations were specifically blocked made it abundantly clear that the CCP and BOCOG were restricting media access to outlets that contradicted the regime's desired narrative (Jacobs, 2008). And there was plenty to hide. Despite their assurances to the IOC, the CCP never intended to improve its human rights record. Quite the contrary, they used the Olympics as a means of ruthlessly suppressing dissent with imprisonment and, in some cases, torture (Cha, 2008). In what looked like a compromise, the BOCOG organized three locations where—they claimed—protest could be carried out away from the sporting events. In order to gain entry, however, protestors needed to apply for a permit. Not only were all 77 permits rejected, a number of potential protestors were incarcerated (Boykoff, 2016). In Tibet, the annual March 10 protests marking the 1959 flight of the Dalai Lama were met with state brutality, with police initiating a crackdown that has stretched to the present. As of the writing of this article, 157 Tibetans have self-immolated to protest the CPC's use of cultural genocide in their homeland (Ross et al., 2021). Uyghurs and other Turkic Muslims, located primarily in the Xinjiang Uyghur Autonomous Region, saw their requests for basic human rights, which were typically labeled as acts of separatism, reframed as threats of terrorism (Roberts, 2020). Today, Xinjiang represents a high-tech penal colony of China (Byler, 2021), with a sprawling system of re-education camps inflicting cultural genocide.

The CCP's unprecedented level of investment in the Olympics was matched only by the magnitude of displacement, estimated to ~1.5 million residents (Centre on Housing Rights Evictions, 2007). As Blunden (2012) explains, “even prior to the bid, redevelopments occurred in order to give Beijing a better chance of winning the bid” (p. 525). The rate of displacement more than doubled when Beijing was elected as an Olympic Host City (Centre on Housing Rights Evictions, 2007). Furthermore, the staggering figure of 1.5 million displaced residents is unlikely to include migrants as government reports typically only included permanent residents who were eligible for compensation (Centre on Housing Rights Evictions, 2007). Although qualitative research has indicated that migrant workers in Beijing were aware of the precariousness of their housing status, had limited attachment to their residences, and did not expect housing rights or compensation, they also recognized that their lives were made harder by displacement (Shin and Li, 2013). For example, being forced further out due to rising house prices and increased travel times to place of work (Shin and Li, 2013). Infringing on human rights, forced evictions in Beijing, and wider China, have traditionally been characterized by arbitrariness, lack of due of process, and external pressure and violence directed toward evictees and their representatives (Centre on Housing Rights Evictions, 2007). In the preparation for the Games, non-migrant residents in the Huijialou region of Beijing, for example, described the deliberate damaging of homes, communities and services (rendering residences and entire areas dangerous and inhabitable), and a range of tactics by hired enforcers that included physical intimidation, harassing residents at night, dumping garbage and defecating in building entryways (Centre on Housing Rights Evictions, 2007). Those who remained in targeted areas often comprised family units that included vulnerable residents, such as the elderly, school-aged children, the chronically ill, and low-skilled unemployed. The reason they gave for remaining was that the inadequate compensation offered would not meet their living costs, lest they move 20–30 km away from the city center (Centre on Housing Rights Evictions, 2007).

## Building the “Repression Ready State”: The London 2012 Olympics

On 6 July 2005, the IOC narrowly declared London, England, the host of 2012 Olympic Games over a very competitive bid from Paris, France, by a final tally of 54–50. The celebrations had barely begun when, the following day, terrorists conducted four, highly coordinated, suicide bombings of London transit, including three in the subway system and a fourth on a bus, taking the lives of 52 innocent people, injuring a further 784. As the London Olympic Games Organizing Committee (LOCOG) prepared for 2012, security was understandably a top priority. When does protecting the people of London, however, transform into something else entirely? As Boykoff and Fussey (2014) explain, the “electrified fences, ubiquitous perimeters, razor wire and enhanced surveillance cameras remain scored into East London's post-Olympics landscape” (p. 266).

Following the human rights disaster that was the 2008 Beijing Olympic Games, the IOC sought to improve its image by formally aligning with the UN, securing Permanent Observer status in 2009 (UN, 19 October 2009). Although the Beijing debacle loamed large, the IOC's Permanent Observer status was a long time coming. The IOC's position as the primary powerbroker of global sport has become increasingly complicated. After substantial boycotts at the 1976 Montreal and 1980 Moscow, and 1984 Los Angeles Olympics, the IOC President Juan Antonio Samaranch sought to strengthen his organization's influence in global sport, and international politics more broadly, by pursuing a much closer relationship with the UN General Assembly (UNGA). As Keys (2017) explains, the irony of such efforts was obvious: “After nearly a century of loudly proclaiming that it was above politics, the IOC now chose to combat ‘politicization' of the Games in the world's most politicized body” (p. 1,162). Beginning in 1995, the UN played along with the IOC's flimsy self-construction as a peace and rights organization, agreeing to “include in the provisional agenda of its fifty-second session the item entitled ‘Building a peaceful and better world through sport and the Olympic Ideal' and to biennialize this item so that it will be considered in advance of each Summer and Winter Olympic Games” (United Nations, 1995). The list of issues the UN and IOC supposedly cooperate on has evolved over time. In 2009, on the eve of the IOC's elevation to Permanent Observer Status, these issues included: “human development, poverty alleviation, humanitarian assistance, health promotion, HIV and AIDS prevention, youth education, gender equality, peacebuilding and sustainable development” (United Nations, 2009a).

While the IOC politicked for Observer Status, UN members raised serious concerns about the 2012 London Olympic Games. Indeed, after meeting from 12 to 13 May 2009, the Committee on Economic, Social, and Cultural Rights (CESCR) expressed concern about a “shortage of adequate stopping sites for Roma/Gypsies and Irish Travelers, and reports concerning evictions of groups of Roma from their sites due to the compulsory purchase order of those sites for the organization of the 2012 Olympic Games in London” (United Nations, 2009c). The IOC was welcomed into the UN fold shortly thereafter, on 19 October 2009. In hegemonic terms, the expansion of the IOC-UN relationship increased the former's coercive potential, aligning it with the very body responsible for formulating, and monitoring adherence to, the *Universal Declaration of Human Rights*. IOC President Jacques Rogge proclaimed: “The Olympic values clearly match with the UN philosophy. Today's decision further strengthens the partnership between the IOC and the UN system” (International Olympic Committee, 2009). UN Secretary-General Ban Ki-moon went so far as to suggest that “Olympic Principles are United Nations Principles.” Although the IOC's stated principles mirrored those of the UN, the body's actions suggested something else entirely.

A broader UNHRC study by Special Rapporteur Raquel Rolnik of Brazil, implicated the Olympic Games in a laundry list of issues, including evictions, gentrification, reduction of social and low-income housing, criminalization of the homeless, and displacement of informal communities (United Nations, 2009d). As Rolnik explains, although the IOC adopted “Olympic Movement Agenda 21,” based on the UN's Agenda 21 for sustainable development, but does not live up to the Agenda's support for the right to adequate housing (United Nations, 2009d). Unfortunately, to quote Rolnik, “Agenda 21…is only a declaratory instrument; hence, the provisions are not readily enforceable. To ensure that practices of the institution are in conformity with housing rights and standards, it is important that they be addressed clearly in binding norms” (p. 13). On 21 December 2009, CESCR member Nicolaas Schrijver of the Netherlands raised more general concern and “wished to know whether the Government was conducting a study on the impact of the future London Olympic Games on human rights, whether institutions had begun a dialogue with human rights defense groups and whether lessons had been learned in that area from the Beijing Games” (United Nations, 2009b, p. 11).

Beijing was not so much a cautionary tale for London as it was a blueprint. Writing on the London 2012 Olympics, Boykoff and Fussey (2014) argue that the games left behind a “repression-ready security state.” This includes visible queues of the London 2012 security legacy, such as electrified fences, surveillance cameras (CCTV), and razor wire, but also a less tangible security inheritance, “organizational innovations integrating civil and military modes of control; new operating standards for physical security; enduring networks of knowledge and practice; streamlined criminal justice responses and the creation of legislative provisions that translate social incivilities into criminal offenses” (p. 266).

Billed as “The People' Games,” London 2012 organizers guaranteed that regeneration, primarily of the working class, industrial East End, would benefit everyone, including the local communities were most redevelopment would occur (Centre on Housing Rights Evictions, 2007; Azzali, 2019). Much of the regeneration was concentrated in six East London “host boroughs,” such as Newham, one of the poorest London Boroughs that contained the second most diverse populations in the UK (70% of residents were non-white) with the youngest age structure of residents in the UK (30% of the population under 20; Kennelly and Watt, 2012). Shaped by austerity politics, the promised legacy failed to materialize in Boroughs like Newham. Instead, it fell short of objectives, and in a pattern remarkably similar to other Games, worsened, rather than resolved issues of inequity and disparity (Bernstock and Davis, 2019). For example, 15 traveler families in Clays Lane, Newham, who had lived there since 1972, and 20 more traveler families from Waterden Road, Hackney, who had lived there since 1993, were removed in the process of state-led displacement (Bernstock and Davis, 2019). In their research, Bernstock and Davis asserted that contrary to a carefully cultivated narrative—that displacement and regeneration in these areas was both necessary and benevolent due to post-industrial dereliction—these were thriving areas with resources and an overt sense of community that would be missed. For the displaced, while relocation processes were rapid, compensation processes were slow and finding alternative housing was hard (Bernstock and Davis, 2019). Adding insult to injury, targets for the creation and occupancy of affordable replacement housing—already severely limited in London—were substantially reduced from original plans, and in some areas, as little as 24% of replacement homes are considered genuinely affordable (Bernstock and Davis, 2019). Privatization and financialization contributed to rapidly escalating housing^5^ and living costs and significant second wave displacement. Such was the extent of displacement, pupils at St Anthony's primary school in Stratford sang songs about their departing classmates (estimated to be as much as half a class per year) (Bernstock and Davis, 2019). Young adults complained of services and opportunities that were neither designed nor catered to them (Kennelly and Watt, 2012), while ethnic analysis of who lived in these constructed homes post-Olympics reinforced claims of exclusion and showed that black and minority groups were grossly underrepresented (Bernstock and Davis, 2019).

The breadth and depth of the Olympic's harmful legacies across the preparations, staging, and aftermath of the Beijing 2008 and London 2012 Games left the IOC in a precarious position. How could an organization trying “to place sport at the service of the harmonious development of humankind, with a view to promoting a peaceful society concerned with the preservation of human dignity” (NOlympics, 2020, p. 11) turn a blind eye to the displacement and securitization associated with their events? How can the UN continue to support such an organization? After the 2012 London Olympics wrapped up, the UN membership's enthusiasm for future Games remained immense. A whopping 119 nations supported the 2013 version of the “Building a peaceful and better world through sport and the Olympic ideal” draft resolution, leaving in such questionable IOC goals as poverty alleviation, humanitarian assistance, health promotion, and sustainable development, all of which are completely incompatible with the IOC's utter lack of real, meaningful human rights protocols for host cities (United Nations, 2013). With such unwavering support in the halls of the progenitor of the Universal Declaration of Human Rights, there can be little question about the IOC's hegemony in the realm of sport and, by extension, the cultural hegemony of the United States and its allies over definitions of appropriate sports hosting, placing grandiose capitalist spectacle ahead of the basic human rights of vulnerable populations.

## The exclusion Games: the Rio Olympics and the Assault on the Poor

In 2015, the UN General Assembly suggested that the IOC could play an important role in its 2030 Agenda for Sustainable Development (United Nations, 2015), consisting of 17 sustainable development goals (SDG), several of which—like “reduced inequalities” and “sustainable cities and communities”—the IOC undeniably exacerbates via the Olympics (United Nations, 2015). The integration of the IOC into the UN fold is here complete. The IOC is now, in a very real and visible way, the sporting arm of the UN. Its integration into the UN system has afforded the IOC with the unjustifiable position of a human rights defender that simply cannot be squared with the realities of hosting their event. Nonetheless, as Black and Hibbeln (2019) explain, “the discourse surrounding Brazil's successful bid stressed the prospective legacy of the Games in decisively addressing the poverty and inequality of the country's past through an emphasis on social development and sustainability in the design of the bid” (p. 661).

It was clear leading up to the 2014 FIFA World Cup that the Brazilian authorities would trample any and all rights and freedoms—including the right to life—to make room for a sporting mega event and maintain order during the fallout. Unfortunately, the UN and IOC were well aware of the human rights violations associated with the 2014 FIFA World Cup preparations and that similar offenses would no doubt precede the Olympics. Amnesty International highlighted the situation in 2011, asking Olympic organizers to urge “Brazilian authorities to stop forcibly evicting hundreds of families across Rio de Janeiro amid preparations for the summer 2016 Summer Olympic Games” (Amnesty International, 2011, para. 1). In 2015, Human Rights Advocates made sure the UN and IOC could not feign ignorance of Amnesty's findings by including their research in a report delivered to the UNHRC. Reiterating Amnesty's plea. Human Rights Advocates explained that “people have been forcefully evicted from their homes in Rio de Janeiro, again without prior notice or consultation, entire communities are fighting imminent eviction, and local businesses have been forced out” (UNHRC, 19 February 2015, p. 3). Neither the UN nor the IOC dealt with the matter in a serious way. The fate of the pair was now firmly connected and neither was willing to risk that relationship, and the continued value of the Olympic Games as vehicle for empty propaganda, over the struggles of thousands of impoverished Brazilians. Indeed, the Olympics were clearly a valuable moment of exception for Rio Mayor Eduardo Paes who infamously quipped: “The Olympics pretext is awesome; I need to use it as an excuse for everything…Some things could be really related to the games, others have nothing to do with them” [as quoted by Keys (2019), p. 171]. Unsurprisingly, as Faulhaber (2020) concludes, the 2016 Rio Olympics ultimately exacerbated the “social and spatial segregation” already evident in the city (p. 223).

Activists labeled the Rio Olympics “the Exclusion Games” (World Cup and Olympics Popular Committee of Rio de Janeiro, 2015). It was a label that reflected the extent to which the Rio Games were mega event, rather than people-orientated, and acted to increase disparities between the affluent and the poorer favela citizens (Schwambach, 2012). Reflective of a past where historical violations of rights to adequate housing are normalized, more than 60,000 people were displaced by Olympic-related building (Faulhaber and Azevedo, 2015). Many received no or low compensation that was inadequate for comparable housing in the local areas, moving them further away (sometimes as much as 40 km) from work and their communities (Silvestre and Gusmão de Oliveira, 2012). Moreover, compensation only referred to costs for the built property and not land value (Silvestre and Gusmão de Oliveira, 2012). Residents were subject to threats, physical intimidation, coercion, service disruption (e.g., halting of electricity and garbage collection) and even demolishment of homes before alternative plans or compensation were in place (Silvestre and Gusmão de Oliveira, 2012; Griffin, 2016). Residents were forced to stay with relatives and friends, move farther away or face homelessness (Silvestre and Gusmão de Oliveira, 2012). In one particularly galling account, a woman's home was demolished while she was at a Doctor appointment—it took 5 months to find her replacement housing (Salvesen, 2015). Ultimately, regeneration (publicly funded) did not benefit displaced residents of regenerated areas, but led to closed, tightly controlled, gentrified, sanitized, neo-liberal, privatized built environments with shopping malls, corporate buildings and gated communities designed for the rising middle-classes and the wealthy (de Quiroz Ribiero and dos Santos Junior, 2017; Azzali, 2019). These environments were often juxtaposed, in stark contrast, against nearby low-income neighborhoods and favelas, reinforcing segregation in the city and how the Rio Games did very little to improve the everyday lives of everyday people (Azzali, 2019).

## Conclusion and Future Directions

We have described how behind the glitz and the glamor, the Olympics leaves a shadow legacy of damage to host cities and vulnerable citizens. From critical perspectives, that spotlight and draw out the ugly side of sport, the Games may appear as an unstoppable destructive juggernaut, destined to wreak havoc on those least able to bear it. Yet, although there is disagreement on how best to go about it, critical social theory from its very beginnings was not developed to serve existing reality (Horkheimer, 2002) but rather open up space and possibility for new ones to emerge (Kellner, 1990). No hegemonic ideology, however deeply entrenched, is ever absolute or beyond reproach or contest (Wright, 1998) and hegemonic consent is never fully realized because it is always contested by actors and groups with competing interests and aims (Kincheloe and McLaren, 2000).

Psychologically, people can have some sense or insight into their domination, or be awoken from a false consciousness, while in relation to historical and temporal forces, there are always remnants of a past that can anchor resistance, transform understandings, and offer the possibility for an emergent future (Gramsci, 2000). Moreover, it stands to reason that with the resources at the disposal of humanity in the modern age, our global society is not the best we can do (Adler et al., 2007). Qualitatively different and better forms of society are possible, particularly because society in its current guise is only the latest in a historical sequence that contains within it the seeds of its own demise (Adler et al., 2007). Such a view is consistent with the ideas within Gramsci's work, which provides, not a static conception of hegemonic dominance of a ruling class, but rather “a society in constant process, where the creation of counterhegemonies remains a live option” (Lears, 1985, p. 571).

Some have already begun to imagine a different Olympics. One option is to establish a permanent, single site, which would offer a “United Olympic Nations of Sport” for training and competition, promoting the global values—like human rights—now encompassed by Olympism and promote real peace through sport (Nauright, 2015). Though radical, it is not unfeasible. In recent years, spiraling costs, blown budgets, white elephant legacies, and subsequent public backlash has significantly dampened enthusiasm for Games hosting (Evans, 2018; Sorkin and Kessler, 2021). There were 11 bids for the 2020 Games, but two for 2024 (Paris) and only one for 2032 (Brisbane). That Tokyo, in a state of COVID emergency, was pinned by an ironclad hosting city contract, and officials left with little option but to push ahead in spite of public and medical outcry over commitment to a pandemic Games, may yet strike a further blow against the previously taken-for-granted, but fast-fading allure of Olympics hosting.

Groups of dedicated activist groups who have, for a number of years now, operated increasingly strategically, have been central to counter-hegemonic incursions that do practical “on the ground” work and that chips away at the once unchallengeable Olympic brand. Boykoff (2021) notes the progress in transnational Olympic organizing that culminated in the first ever anti-Olympics summit in Tokyo 2019, replete with strategy sharing, public talks and mobilization of local districts, community members and activist groups from past, previous and future Olympic host cities. Boykoff (2021) goes onto explain the shift from activism as a *moment* of movements, whereby a central entity in a host city steers local activist groups under a temporary umbrella while the Games are in town, with energy and focus dissipating when the Games are over) to a *movement* of movement (marked by increased transnational cooperation on a global scale) that transcends a single event.

Academics, in the image of Gramsci's “organic intellectual” also have a central role in such on the ground activism as well as in their research and writing endeavors, through which they can encourage the broader public to share in a more critical and skeptical view of the Olympic hegemonic order and Pac Americana, or what may come to replace it. Here, we may think and be inspired by the famous dictum associated with Gramsci's “Pessimism of the intellect, optimism of the will” (1917) where the hearts and minds of the majority can be won and any position or influence conferred by academics ought to also be harnessed to form alliances with a broad-range of social strata (Parry, 1984). We propose that central to both of these tasks is the creation of compelling, resonant narratives. Drawing on meaning-making processes in cultural sociology and entrepreneurship, these are the narratives constructed from the available cultural repertoire of actors and the institutional field to shape the “attention and perceptions of targeted others” (Wry et al., 2011, p. 450). The construction of such narratives by the critical scholarly community, acting as skilled cultural operators, can transcend the goal of making facts and information salient, and resonate with and shape what audiences take to be real, meaningful and important (Soublière and Lockwood, 2018; Lounsbury and Glynn, 2019), propelling people to action and supporting a transformation in critical consciousness that has always been at the strategic heart of critical theory. Such a move holds the possibility of actually refashioning the Olympics into something of integrity and beauty, and that can act in service of humanity, as is so frequently claimed.

## Data Availability Statement

The raw data supporting the conclusions of this article will be made available by the authors, without undue reservation.

## Author Contributions

Both authors listed have made a substantial, direct, and intellectual contribution to the work and approved it for publication.

## Conflict of Interest

The authors declare that the research was conducted in the absence of any commercial or financial relationships that could be construed as a potential conflict of interest.

## Publisher's Note

All claims expressed in this article are solely those of the authors and do not necessarily represent those of their affiliated organizations, or those of the publisher, the editors and the reviewers. Any product that may be evaluated in this article, or claim that may be made by its manufacturer, is not guaranteed or endorsed by the publisher.

## Footnotes

^1^We stop short of using the language *Pax Britannica* and *Pax Americana*. The terms feel too reductionist given the grand machinations within global hegemony.

^2^The original French reads: “*développe également une diplomatie expansionniste destinée à étendre l'empire olympique dans un double jeu de séduction commerciale et d'allégeance politique vis-à-vis des institutions internationales (Onu, G8, Unesco, etc.), des bailleurs de fonds (FMI, Banque mondiale) et des grandes puissances, et tout particulièrement des nations émergentes ou de ce que l'on nomme maintenant le* ≪ *Bric* ≫ *(Brésil, Russie, Inde, Chine)*.”

^3^ASPIRE is an acronym for “Attitude, Sportsmanship, Pride, Individual responsibility, Respect, Express yourself.” See, “Australia/ASPIRE School Network,” https://olympics.com/ioc/olympic-values-and-education-program/initiatives/australia-aspire-initiatives.

^4^The notion of the Olympics using Trojan Horses, in terms of neo-liberalism, has also been used by Zirin (2012). See Zirin's interview with *Democracy Now!*, 19 June 2014. https://www.democracynow.org/2014/6/19/a_neo_liberal_trojan_horse_dave.

^5^For example, in Chobham Manor, the first entirely new neighborhood, 72% of homes were for market and ranged between £465,000 and 859,000 (~$587,000–1,084,370; (Bernstock and Davis, 2019).
